# Drug development targeting the mitochondrial respiratory chain of *Sparganum proliferum*: Initial biochemical and drug discovery insights into the enigmatic helminth parasite

**DOI:** 10.1371/journal.pntd.0014662

**Published:** 2026-08-19

**Authors:** Shigehiro Enkai, Yurie Nakano, Madoka Koyanagi, Yutaka Arimura, Hirotaka Kanuka, Kenji Ishiwata

**Affiliations:** 1 Department of Pediatrics, Teikyo University School of Medicine, Tokyo, Japan; 2 Asia International Institute of Infectious Disease Control, Teikyo University School of Medicine, Tokyo, Japan; 3 Department of Tropical Medicine, The Jikei University School of Medicine, Tokyo, Japan; 4 Host Defense for Animals, School of Animal Science, Nippon Veterinary and Life Science University, Tokyo, Japan; National Autonomous University of Mexico, UNITED STATES OF AMERICA

## Abstract

*Sparganum proliferum* undergoes asexual proliferation within the human host, leading to multiorgan failure and death. Currently, no effective treatment is available. Long considered mysterious, the natural host and transmission route remain unidentified, hindering preventive measures. Furthermore, owing to its extreme rarity, biochemical research and drug development have been neglected. This study investigated mitochondrial function and screened for compounds targeting this parasite. The parasite showed activity of mitochondrial complexes I–IV and NADH-fumarate reductase, indicating a hybrid respiratory chain that supports both aerobic and anaerobic respiration. Quinone-binding site inhibitors showed inhibitory activity against the respiratory chain. Ascofuranone derivatives acted as dual inhibitors of complexes II and III. The antimalarial drug atovaquone inhibited complex III at a very low concentration (IC₅₀ 2.2 nM). IACS-010759 potently inhibited complex I (IC₅₀ 16.1 nM), causing worm body swelling, surface destruction, and mitochondrial morphological changes in culture assays. Further investigation of the mitochondrial respiratory chain of *S. proliferum* to develop targeted candidate drugs is warranted.

## Author summary

*Sparganum proliferum* is a rare but devastating parasite. Unlike typical tapeworms, it reproduces uncontrollably in the human body, invading organs like the brain, liver, and skin. With no known cure and a largely mysterious lifecycle, infections are almost always fatal. This study aimed to identify a biological weak point to guide future drug development, focusing on the parasite’s mitochondria. We discovered that it is a metabolic hybrid, capable of both aerobic and anaerobic respiration to generate energy. This flexibility may let it thrive in various body sites. We tested compounds targeting mitochondrial function. Atovaquone, an existing antimalarial, potently blocked the respiratory chain at low concentration. IACS-010759, a mitochondrial complex I inhibitor, inhibited complex I of this parasite at low concentrations, and culture tests confirmed structural alterations of both its body surface and mitochondria. These results highlight the respiratory chain as a promising therapeutic target. Though more work is needed to establish human safety and efficacy, this study provides the first biochemical roadmap for treating this deadly helminthic infection.

## Introduction

**Fig 1:**
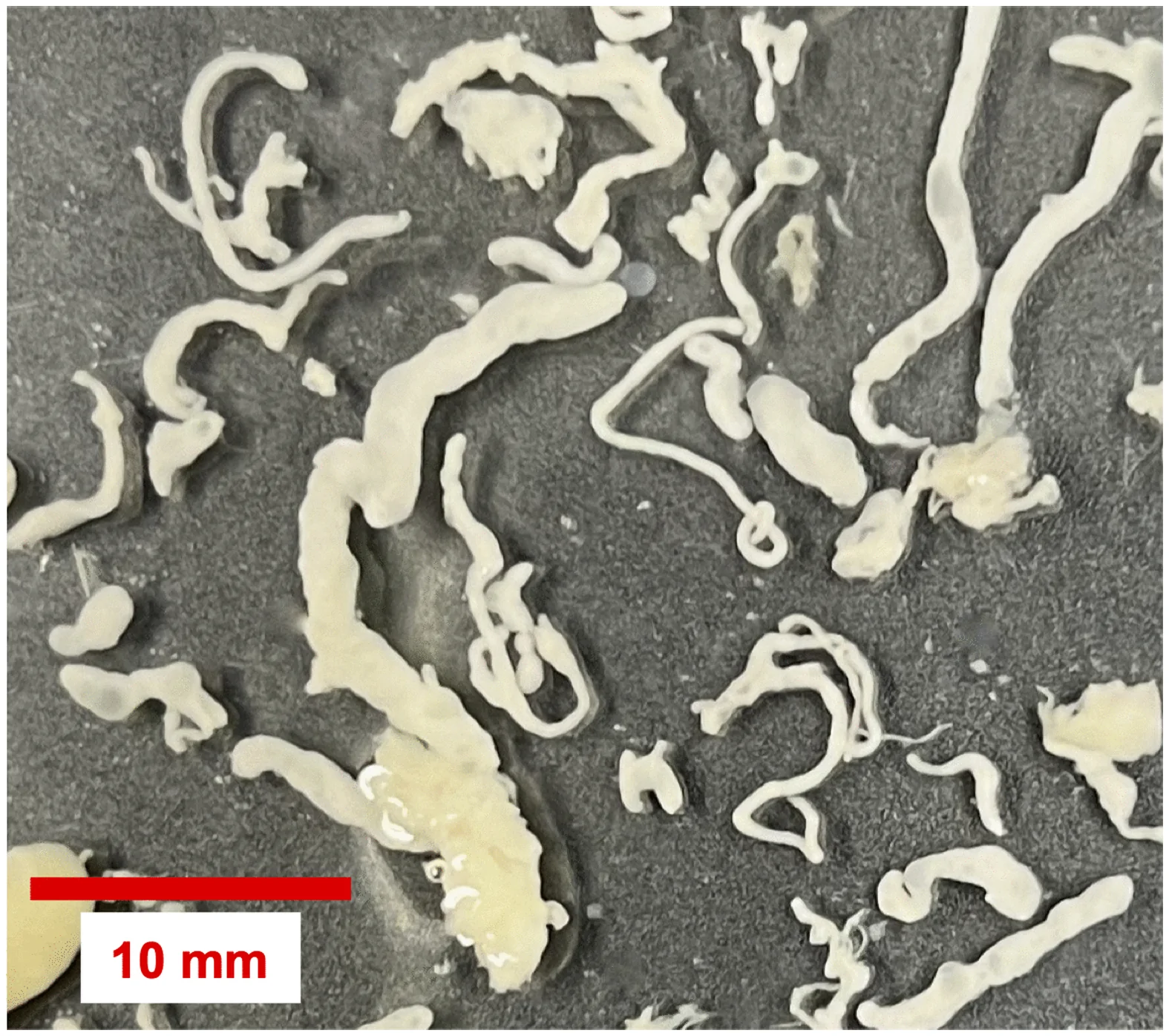
Sparganum proliferum is characterized by plerocercoids measuring 2–5 cm in length with variable thickness. The body surface is smooth and lacks both oral and excretory apertures. The branched morphology has been described as the “medusa form.” The definitive host and transmission route remain unknown, and it has been hypothesized that this parasite lacks an adult stage. In human infections, the larvae undergo uncontrolled asexual proliferation within multiple organs and subcutaneous tissues. Due to the absence of effective therapeutic interventions, such infections are invariably fatal.

Human proliferative sparganosis is caused by *Sparganum proliferum,* a pseudophyllidean tapeworm (Fig 1), and it is one of the most harmful and fatal parasitic diseases [1]. A total of 18 cases of this parasitosis, including 2 suspected cases, have been reported since 1905 in Asia and North and South America [2]. Patients infected with this parasite present with extremely severe clinical symptoms, leading to profoundly debilitating and distressing outcomes. During the clinical course, *S. proliferum* larvae reproduce asexually and indefinitely within the human body. These larvae subsequently spread to and invade various organs and tissues, including the lungs, liver, skin, bones, and brain, ultimately causing multiorgan failure and death.

Human proliferative sparganosis consists of two disease types, cutaneous and internal [2]. The cutaneous type presents with dermal manifestations with multiple nodules covering large skin areas due to invasion by the parasite. In the internal type, nodules or masses are formed by the invasion of worms into internal organs and tissues. The asexual proliferation of the parasite and dissemination throughout the whole body make it difficult to cure surgically. In both types, the larvae demonstrate unrestrained proliferation accompanied by relentless migration throughout the host tissues, thereby inducing progressive and irreversible destruction of visceral organs and neural structures, ultimately resulting in a fatal outcome for the patient. However, an effective antiparasitic drug for *S. proliferum* remains to be found, despite the patients’ miserable prognosis.

It has been reported that *S. proliferum* is a close relative of *Spirometra erinaceieuropaei* based on mitochondrial CO1 gene analysis [3,4]. Since *S. erinaceieuropaei* does not proliferate asexually in humans, it causes non-life threatening sparganosis, just larva migrans, unlike *S. proliferum*. The lifecycle of *S. erinaceieuropaei* has been well described. Humans are generally infected by ingestion of paratenic hosts such as crustaceans or second intermediate hosts such as fish and reptiles with the larvae. In contrast, both the adult and larval stages of *S. proliferum* have not been observed in wildlife at all. In 2023, great progress was made in conducting whole-genome sequence analysis with the larvae isolated from a Venezuelan patient [5]. Interestingly, the genome analysis showed that *S. proliferum* lost the ability to complete the sexual life cycle, indicating that the parasite may continue to infect and proliferate while remaining in the larval stage without developing to the adult stage [5,6]. The life cycle of *S. proliferum* is unknown, which means that infection control measures cannot be established based on the infection route. The uncertainty surrounding the route of infection underscores the significance of administering appropriate treatment at the time of infection.

**Fig 2:**
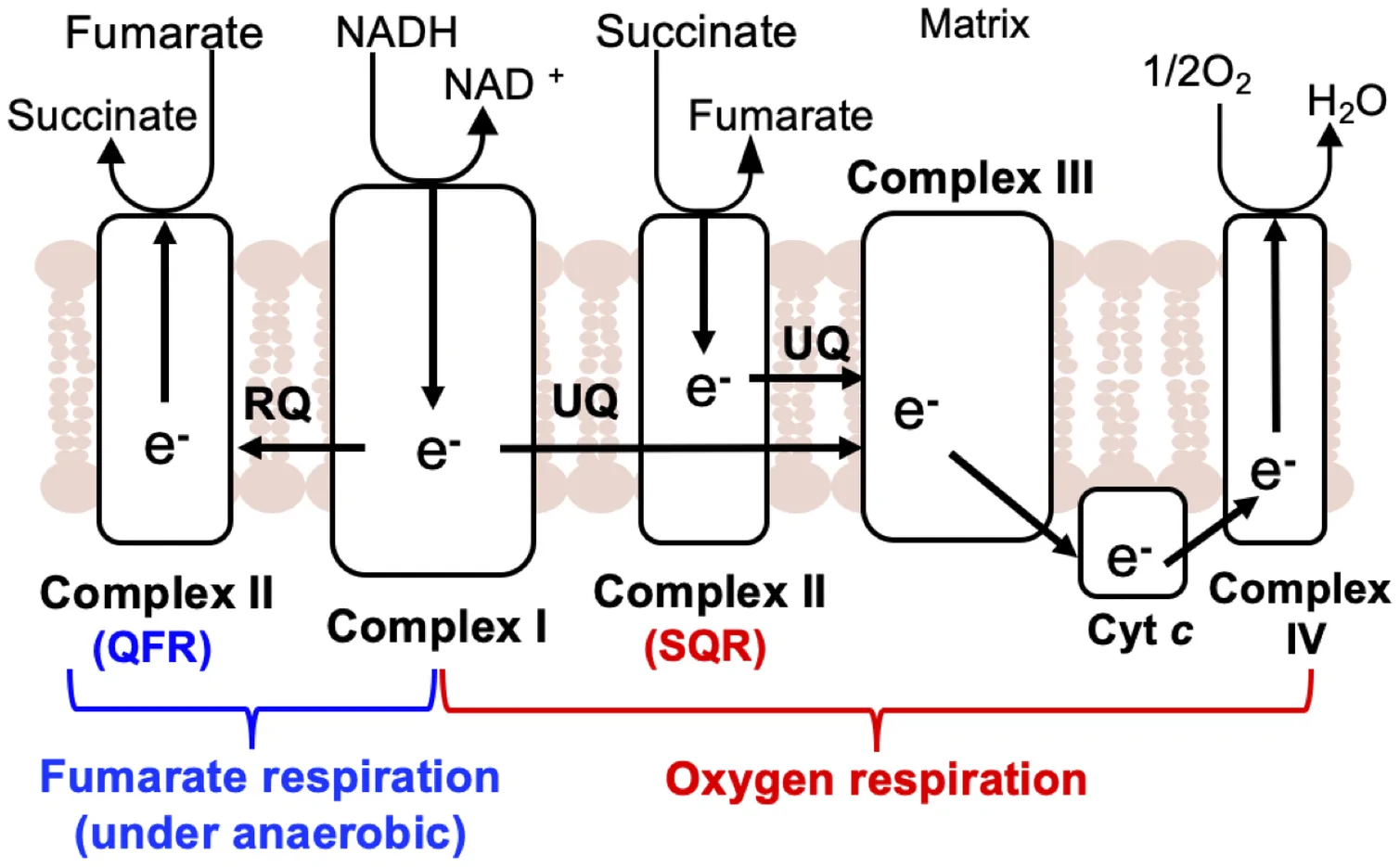
An overview of the mitochondrial respiratory chain. Oxygen respiration engages complexes I, II, III, and IV, with ubiquinone (UQ) serving as the electron carrier between complexes I, II, and III and cytochrome *c* (Cyt *c*). Complex II functions as a succinate-quinone reductase (SQR), transferring electrons from succinate to UQ. Ultimately, electrons originating from both NADH and succinate are used to reduce molecular oxygen, culminating in the formation of water. In contrast, fumarate respiration, mediated by the NADH-fumarate reductase system, encompasses the involvement of complex I, rhodoquinone (RQ), and complex II, also known as quinol-fumarate reductase (QFR). Within this pathway, electrons derived from NADH are initially transferred to RQ via complex I, followed by their subsequent transfer to fumarate through the QFR activity of complex II.

Many aspects of the pathology of this parasite remain to be elucidated. As the first step to elucidate the biochemical features of this parasite, we focused on the mitochondrial respiratory enzymes of the parasite. Mammalian hosts use oxidative phosphorylation through the mitochondrial electron transport chain known as the respiratory chain, which consists of complexes I–IV (Fig 2). Parasites change their energy metabolism to adapt to environmental changes in their host. *Ascaris suum*, a pig roundworm, for example, switches oxidative phosphorylation to fumarate respiration, the NADH-fumarate reductase system, which is anaerobic respiration, when residing in the hypoxic environment of the host’s small intestine in the adult stage [7,8]. Fumarate respiration consists of mitochondrial complexes I and II. Electrons from NADH are received by rhodoquinone through mitochondrial complex I and then transferred to fumarate via the quinol-fumarate reductase activity of mitochondrial complex II under hypoxia, which contributes to the production of ATP without oxygen (Fig 2). In contrast, Fasciola flukes, and *Echinococcus multilocularis*, a cyclophyllidean tapeworm, switch between oxidative phosphorylation and fumarate respiration depending on the oxygen environment in the colonized organs [9,10]. Complexes I to III on these mitochondrial respiratory pathways have been reported to be the targets of quinone binding site inhibitors, leading to the potential development of novel antiparasitic agents [10–15]. However, regarding *S. proliferum*, even the most foundational information remains entirely unavailable, both in terms of its basic mitochondrial biochemical properties and its viability as a drug target. Based on these considerations, we hypothesized that the mitochondrial respiratory chain of *S. proliferum* represents a viable and exploitable drug target for the development of novel therapeutic agents against this otherwise untreatable infection.

The activities of mitochondrial complexes I, II, III, and IV were investigated using mitochondria prepared from the larval stage of *S. proliferum*. Furthermore, the inhibitory effects of representative quinone-binding site inhibitors on the mitochondrial complexes of *S. proliferum* were examined, and the parasite-killing abilities of the compounds were evaluated. This study provides novel insights into the characteristic mitochondrial functions of *S. proliferum* and the potential for developing drugs to treat infections with this parasite.

## Materials and methods

### Animal ethics statement

All animal experiments were reviewed and approved by The Institutional Animal Care and Use Committee of Jikei University (Approval No. 2020–036). All procedures were performed in strict accordance with the national guidelines for animal experiments in Japan and the Jikei University regulations on animal experimentation.

### Materials

The Venezuela strain of *S. proliferum*, originally isolated from a Venezuelan patient in 1981, provided by Dr. T. Kuramochi at the National Science Museum, was used in this study. The strain has been maintained by serial passages using Slc: ICR mice at The Jikei University School of Medicine. The *S. proliferum* used in this study is the same strain used in the genome projects reported by Kikuchi et al. [5]. The parasites were recovered from mice at 10 months post-infection and used for the experiments.

### Preparation of the mitochondrial fraction from *S. proliferum*

The enriched mitochondrial fraction of *S. proliferum* was prepared based essentially on the method for *Echinococcus multilocularis* [10,11]. Briefly, the parasite materials were homogenized with a motor-driven homogenizer (six passes, three to four times) (AS ONE, Osaka, Japan). The homogenate was diluted with the mitochondrial preparation buffer [210 mM mannitol (Sigma, St. Louis, MO, USA), 10 mM sucrose (Sigma), 1 mM disodium EDTA (Sigma), and 50 mM Tris-HCl [pH 7.5] (Sigma)] supplemented with 10 mM sodium malonate (Sigma) to 5 times the volume of the original parasite sediment, and then centrifuged at 800 × *g* for 10 min (4 °C) to precipitate cell debris and nuclei. The supernatant was then centrifuged at 8,000 × *g* for 10 min (4 °C) to obtain the mitochondrial pellet. The pellet was resuspended in mitochondrial preparation buffer (without malonate) and centrifuged at 8,000 × *g* for 10 min (4 °C). The enriched mitochondrial fraction was suspended in mitochondrial preparation buffer without malonate. The enriched mitochondrial fraction yielded approximately 1 mg of protein from 5 g of wet worm tissue.

### Protein quantification

Protein concentrations were determined using the Pierce 660 nm Protein Assay Kit (Sigma-Aldrich, St. Louis, MO, USA) according to the manufacturer’s instructions. Briefly, 10 μL of each standard and sample solution were dispensed into a 96-well plate, followed by the addition of 150 μL of the assay reagent to each well. After incubation for 5 min at room temperature, absorbance was measured at 655 nm using an iMark microplate reader (Bio-Rad Laboratories, Hercules, CA, USA).

### Enzyme assays

All enzyme assays using mitochondrial fractions were performed in 1-mL reaction mixtures at 25 °C. The mitochondrial fractions were thawed at room temperature and then returned to a deep freezer for refreezing before the assay. One freeze-thaw cycle was performed to make the mitochondrial membrane permeable to the solutes. Succinate dehydrogenase (SDH), NADH-quinone reductase, succinate-quinone reductase (SQR), succinate-cytochrome *c* reductase, NADH-cytochrome *c* reductase, NADH oxidase, and NADH-fumarate reductase activities were measured using a UV-3000 spectrophotometer (Shimadzu, Kyoto, Japan), as described previously [10,11]. The reagents used in each assay were mixed with the reaction buffer [30 mM potassium phosphate (Sigma), 1 mM MgCl_2_ (Sigma), pH 7.5]. The final concentration of mitochondrial protein was 50 μg/mL of reaction mixture. SDH activity was measured by the absorbance change of 2-(4,5-dimethyl-2-thiazolyl)-3,5-diphenyl-2H-tetrazolium bromide [MTT; 60 μg/ml (Sigma)] at 570 nm (ε = 17 mM^-1^ cm^-1^) in the presence of 120 μg/ml phenazine methosulfate (Sigma) and 2 mM potassium cyanide (KCN; Sigma). The reaction was started by the addition of 10 mM succinate (Sigma) to the mixture. NADH-quinone reductase activity (complex I) was measured in 50 mM potassium phosphate buffer (pH 7.4) containing 2 mM KCN, and 60 μM decylubiquinone (dUQ; Sigma). The activity assay was started by adding 50 μM NADH (Fujifilm Wako, Tokyo, Japan) and monitoring the absorbance change of reduced NADH at 340 nm (ε = 6.2 mM^-1^ cm^-1^). The reagents used in the SQR activity assay (complexes I and II) were mixed with the reaction buffer containing 50 mM potassium phosphate (pH 7.4) and 0.1% (wt/vol) sucrose monolaurate (DOJINDO, Kumamoto, Japan). SQR activity was determined by monitoring the change in absorbance of quinone at 278 nm (ε = 15 mM^-1^ cm^-1^) in the presence of 60 μM dUQ and 2 mM KCN. The reaction was initiated by the addition of 10 mM disodium succinate to the mixture. Succinate-cytochrome *c* reductase activity (complexes II and III) was detected by monitoring the absorbance change of reduced cytochrome *c* at 550 nm (ε = 19 mM^-1^ cm^-1^) in the presence of 50 μM cytochrome *c* and 2 mM KCN. The reaction was initiated by the addition of 10 mM disodium succinate to the mixture. NADH-cytochrome *c* reductase activity (complexes I and III) was determined by the same method as used for succinate-cytochrome *c* reductase activity assay in the presence of 100 mM sodium-malonate, 50 μM cytochrome *c*, and 2 mM KCN. The reaction was started by the addition of NADH to a final concentration of 50 μM. NADH oxidase activity (complexes I and Ⅳ) in the isolated mitochondrial fraction was determined in the presence or absence of 2 mM KCN, 100 mM malonate, or both by measuring the absorbance of NADH at 340 nm. The reaction was initiated by the addition of 100 μM NADH to the mixture. NADH-fumarate reductase activity, the anaerobic energy pathway, was determined by the absorbance change of NADH. The reaction medium was supplemented with 100 μg/ml glucose oxidase (Sigma), 2 μg/ml catalase (Sigma), and 10 mM β-D-glucose (Sigma) and left for 3 min to achieve the anaerobic condition. Enzyme activity was determined by monitoring the oxidation of 100 μM NADH at 340 nm. The reaction was started by the addition of 5 mM fumarate (Sigma).

### Determination of 50% inhibitory concentration (IC_50_) values

The 50% inhibitory concentration (IC_50_) values of representative complex I, II, and III inhibitors against the specific activities of mitochondrial respiratory enzymes in *S. proliferum* were determined [10]. Each inhibitor was screened at a concentration of 10 and 25 μM, and IC₅₀ values were determined for compounds exhibiting an inhibition rate greater than 50%. The IC_50_ of each compound was determined by calculating approximation lines from three or more points on either side of the concentration causing 50% inhibition. Rotenone (Sigma), IACS-010759 (MedChemExpress, Monmouth Junction, NJ, USA), metformin (FUJIFILM Wako), pyrantel pamoate (FUJIFILM Wako), pyrvinium pamoate (FUJIFILM Wako), atpenin A5 (Cayman Chemical, Ann Arbor, MI, USA), ascofuranone and its derivative (Faculty of Engineering, Tottori University, Tottori, Japan), atovaquone (FUJIFILM Wako), and antimycin A (FUJIFILM Wako) were tested as the inhibitors of the representative quinone binding sites in the assays. The IC_50_ values of atpenin A5 and ascofuranone for complex III were determined on the basis of NADH-cytochrome *c* reductase activity (complexes I and III), because succinate-cytochrome *c* reductase activity (complexes II and III) was inhibited by their potent inhibitory effect on complex II.

### In vitro experiment of living *S. proliferum*

Parasite culture was performed using RPMI-1640 medium (FUJIFILM Wako) supplemented with 5% newborn calf serum (Gibco, Thermo Fisher Scientific, Waltham, MA, USA), 100 IU/mL penicillin, and 100 µg/mL streptomycin (Sigma). Parasites were harvested from mice 6 months after infection and used for the experiments. For the experimental group, IACS-010759 was added to a final concentration of 50 µM. The control group received dimethyl sulfoxide (DMSO) (Sigma). For each group, eight worms were used, and the experiment was repeated twice. Morphological characteristics were independently verified by two observers. Parasites recovered from mice were cultured for two weeks under either aerobic conditions (5% CO_2_, 37 °C) or anaerobic conditions. In the anaerobic experiments, parasites were sealed in a plastic container with an oxygen scavenger (AnaeroPack-Kenki, Mitsubishi Gas Chemical Company, Tokyo, Japan) to maintain oxygen concentrations below 0.3% at 37 °C.

### Preparation of specimens for optical microscopy

The parasites were fixed in 4% paraformaldehyde PBS, embedded in paraffin, sectioned, and then stained with hematoxylin-eosin (HE) according to the standard protocol [16].

### Preparation of specimens for transmission electron microscopy

For electron microscopy observation, the sample was prepared based on the previously reported procedure [17]. Briefly, the parasites were fixed overnight at 4 °C in half-strength Karnovsky’s fixative (2% PFA and 2.5% glutaraldehyde in 0.1 M phosphate buffer, pH 7.3), followed by postfixation in 1% osmium tetroxide in the same buffer for 2 hours at 4 °C. Ultrathin sections were prepared using a diamond knife and stained with uranyl acetate and lead citrate. Observations were performed using a JEOL JEM-1400Plus transmission electron microscope (JEOL Ltd., Tokyo, Japan).

### Statistical analysis

Descriptive statistics, including the calculation of means and standard deviations (SD) from three independent experiments, were performed using Microsoft Excel version 16.0 (Microsoft, Redmond, WA, USA).

## Results

### Enzyme activities of *S. proliferum* mitochondria

Table 1 presents the specific enzymatic activities associated with the mitochondrial aerobic and anaerobic respiratory chains in *S. proliferum*. Succinate dehydrogenase (SDH) activity was 72.1 nmol/min/mg and was inhibited by malonate. The activity of NADH-quinone reductase (complex I) was 41.1 nmol/min/mg, whereas that of succinate–quinone reductase (SQR; complex II) activity was 145 nmol/min/mg. NADH-cytochrome *c* reductase activity (complex I to complex III) was 22.1 nmol/min/mg, and succinate-cytochrome *c* reductase activity (complex II to complex III) was 29.0 nmol/min/mg, indicating comparable levels of these two activities. NADH oxidase activity (complex I to complex IV) was 16.4 nmol/min/mg and was inhibited by KCN. Furthermore, anaerobic respiratory chain activity, as assessed by NADH-fumarate reductase activity, was 37.5 nmol/min/mg under anaerobic conditions.

**Table 1:** The specific activity of respiratory enzymes in the mitochondrial fraction isolated from *S. proliferum.*

Assay	Sp act* (nmol/min/mg of protein)	
	(mean SD)	
Succinate dehydrogenase (SDH)	complex II	72.1 ± 1.0
SDH with malonate (100 mM)		2.2 ± 0.8
NADH-quinone reductase	complex I	41.1 ± 8.8
Succinate–quinone reductase (SQR)	complex II	145.0 ± 7.0
NADH-cytochrome *c* reductase	complex I -III	22.0 ± 2.1
Succinate-cytochrome *c* reductase	complex II - III	29.0 ± 2.6
NADH oxidase	complex I - IV	16.4 ± 3.9
NADH oxidase with KCN		2.1 ± 0.5
NADH-fumarate reductase (anaerobic)	complex I - II	37.5 ± 5.9

NADH-quinone reductase (complex I): 50μM NADH as a substrate in the presence of 2 mM KCN. SDH and SQR: 10 mM succinate as a substrate. NADH-cytochrome c reductase (complex I and III) and succinate-cytochrome *c* reductase (complex II and III): NADH and succinate were used as substrates, respectively, with KCN or other respiratory inhibitors applied to block alternative pathways as described in the Methods. *Specific activities (Sp act) were determined from at least three independently isolated mitochondria.

### Effects of quinone-binding site inhibitors on the mitochondrial respiratory chain of *S. proliferum*

**Fig 3:**
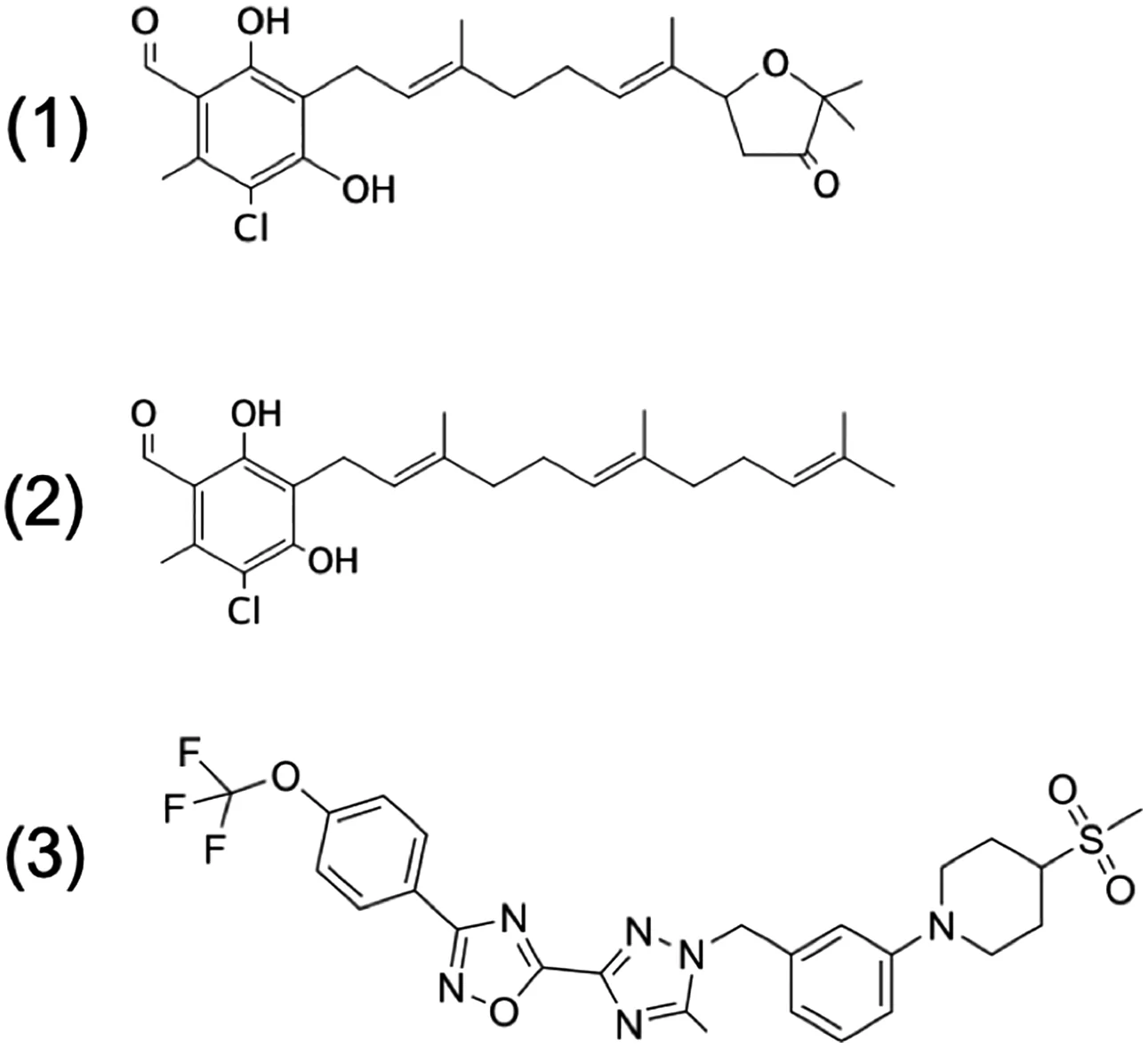
Structures of quinone binding site inhibitors: (1) ascofuranone (AF), an inhibitor of trypanosome alternative oxidase and mitochondrial complex II; (2) AF derivative 1; and (3) IACS-010759, a mitochondrial complex I inhibitor.

The inhibitory activities of various compounds targeting mitochondrial complexes were evaluated by determining their IC_50_ values (Table 2). The complex I inhibitors rotenone and IACS-010759 (Fig 3) demonstrated potent inhibition of complex I, with IC_50_ values of 11.5 nM and 16.1 nM, respectively, while exhibiting no inhibitory effects on complexes II or III. In contrast, metformin, pyrantel pamoate, and pyrvinium pamoate did not exhibit any inhibitory activity against the mitochondrial respiratory chain in *S. proliferum*. Of the complex II inhibitors, atpenin A5, ascofuranone, and ascofuranone derivative 1 (Fig 3) inhibited complex II with IC_50_ values of 6.1 nM, 1447 nM, and 86.8 nM, respectively. Notably, ascofuranone and its derivative also inhibited complex III, with IC_50_ values of 60.7 nM and 42.5 nM, respectively. The inhibitory effects of atovaquone and antimycin A on complex III were also confirmed; atovaquone inhibited complex III with an IC_50_ value of 2.2 nM and complex II with an IC_50_ value of 4600 nM, whereas antimycin A inhibited complex III with an IC_50_ value of 0.77 nM.

**Table 2:** Inhibitory effects of representative quinone-binding site inhibitors on *S. proliferum* (IC_50_ nM).

Compounds	NADH-quinone reductase	Succinate–quinone reductase	NADH-cytochrome *c* reductase
	(complex I)	(complex II)	(complex I – III)
Rotenone	11.5 ± 3.5	>25000	>25000*
IACS-010759	16.1 ± 8.3	>25000	>25000*
Metformin	>25000	>25000	>25000
Pyrantel pamoate	>25000	>25000	>25000
Pyrvinium pamoate	>25000	>25000	>25000
Atpenin A5	>25000	6.1 ± 0.7	>25000
Ascofuranone (AF)	>25000	1447 ± 300	60.7 ± 9.2
AF derivative 1	>25000	86.8 ± 14.9	42.5 ± 7.5
Atovaquone	>25000	4600 ± 1200	2.2 ± 0.6
Antimycin A	>25000	>25000	0.77 ± 0.02*

IC_50_ values for rotenone, IACS-010759, and antimycin A were determined using *succinate-cytochrome *c* reductase (complex II - III). Values for metformin, pyrantel pamoate, pyrvinium pamoate, atpenin A5, ascofuranone and its derivative 1, and atovaquone were determined using NADH-cytochrome *c* reductase (complex I - III).

### Effects of a complex I inhibitor, IACS-010759, on living *S. proliferum* in culture

**Fig 4:**
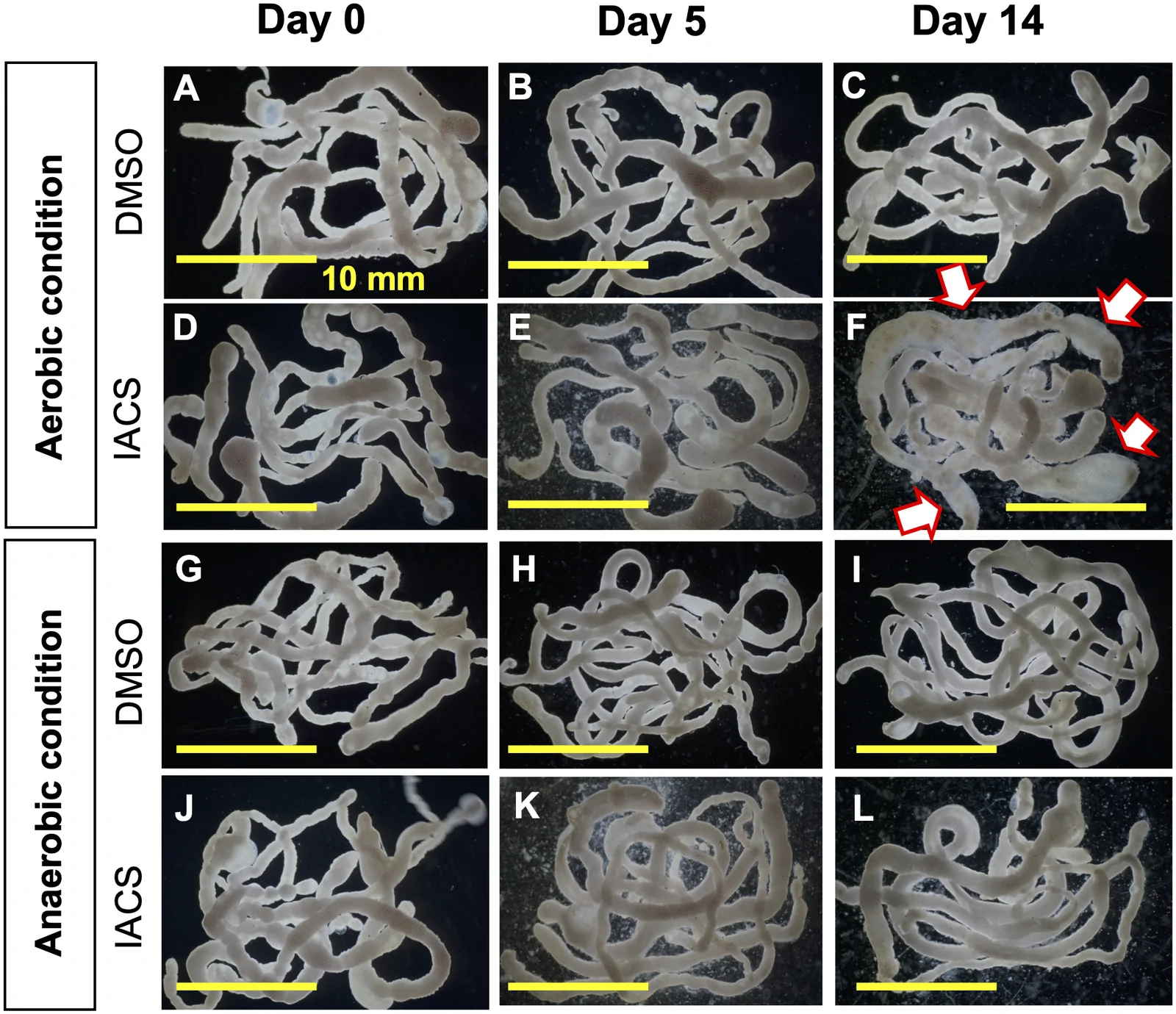
Effects of a complex I inhibitor, IACS-010759, on *S. proliferum* in culture. Morphological changes of the worms and sediment are more noticeable in aerobic than in anaerobic conditions. The arrows indicate the structurally deformed parts of the worms. A-F: aerobic culture condition, G-L: anaerobic culture condition. A-C and G-I: DMSO treatment, D-F and J-L: IACS-010759 treatment. All bars indicate 10 mm.

Morphological changes in cultured parasites treated with IACS-010759, a potent inhibitor of mitochondrial complex I, were observed. The surface of the living worms was smooth and intact at the start of culture. No significant changes were observed in either aerobic or anaerobic culture following DMSO treatment throughout the experimental period (Fig 4A–4C and Fig 4G–4I). However, in parasites cultured in IACS-supplemented medium, subtle alterations in the boundary began to appear as early as day 1 of culture, which advanced progressively and became distinct by day 5 (Fig 4E, 4F, 4K and 4L). By day 14, this had advanced to pronounced structural deformation of the worm body. By day 5 of IACS treatment, turbidity and sediment were observed in the culture medium of both aerobic and anaerobic cultures, accompanied by progressive boundary disruption on the worm surface (Fig 4E and 4K). The surface irregularity gradually became granular, with detached particles and fine granular material leaking from the disrupted surface accumulating as sediment. Sediment was removed during medium changes. By day 5 of aerobic culture, both the surface irregularity and sediment volume had increased further (Fig 4E). By day 14, structural deformation was observed in the remaining worms under aerobic conditions (Fig 4F). Under anaerobic culture conditions, whereas boundary disruption and sedimentation were also observed following IACS treatment, these changes appeared to progress more slowly than with aerobic culture, and no structural collapse was observed by day 14 (Fig 4L). It should be noted that definitive determination of parasite viability could not be made based on these morphological observations alone.

### Morphological observation of *S. proliferum* co-culture with IACS-010759

**Fig 5:**
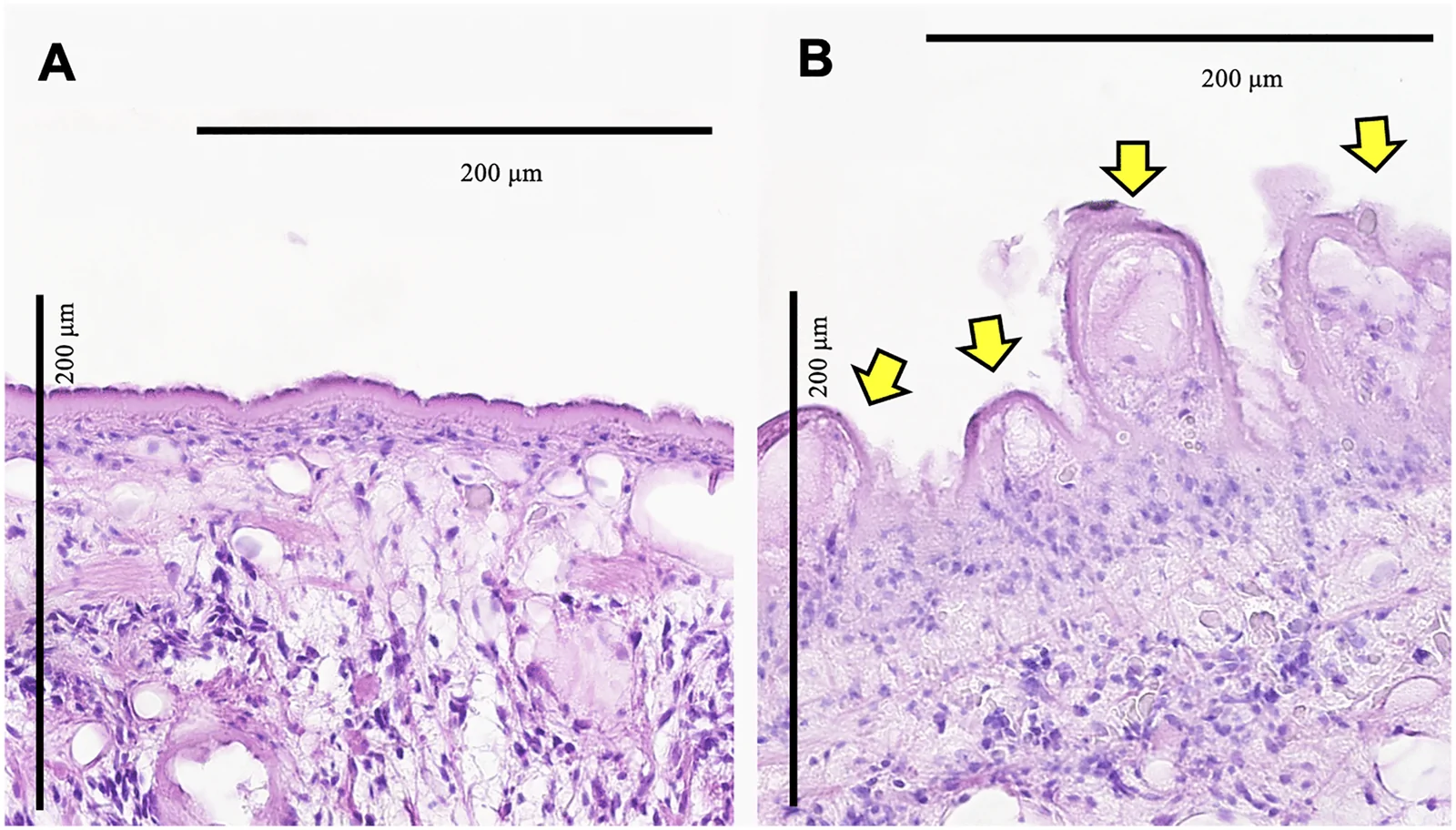
Effects of a complex I inhibitor, IACS-010759, on *S. proliferum* in culture. Under anaerobic conditions, partial swelling of the lower part of the tegument in the worm (arrows) is observed on day 1 with IACS treatment. A: DMSO treatment, B: IACS treatment. Bars indicate 100 μm (20 × objective).

**Fig 6:**
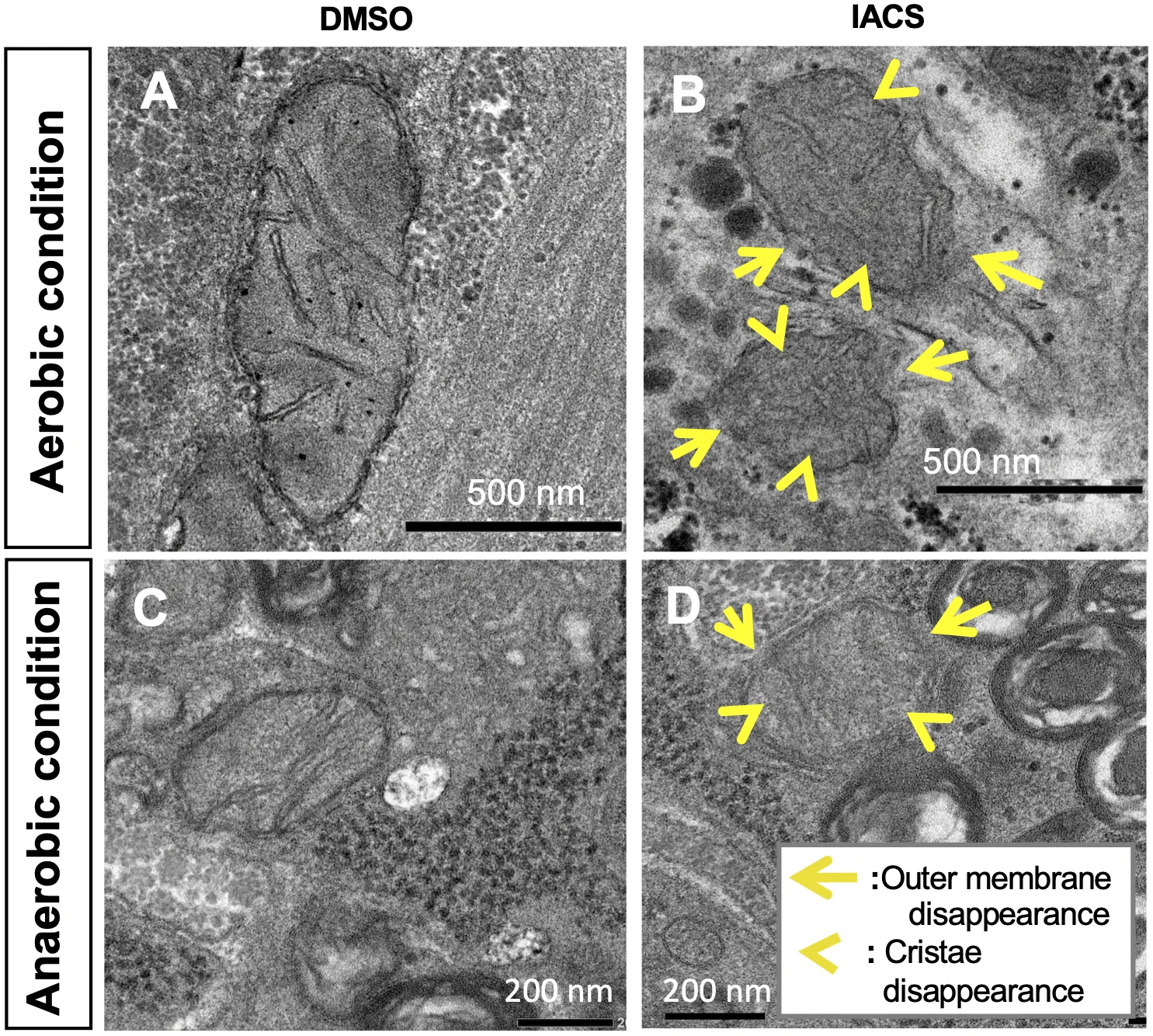
Effects of a complex I inhibitor, IACS-010759, on *S. proliferum* in culture on day 1. In some IACS-treated mitochondria, disappearance of the outer membrane (arrows) and cristae (arrowheads) is observed. A & B: aerobic condition, C & D: anaerobic condition. A & C: DMSO treatment, B & D: IACS treatment. Bars indicate 500 nm for A & B, and 200 nm for C & D.

Body surface disruption was observed under aerobic conditions but was unidentifiable under anaerobic conditions on Day 1 (textual records only). Consequently, the body surface of the anaerobic parasites was further evaluated via optical microscopy. The effect of IACS in anaerobic culture was not as pronounced as in aerobic culture, but optical microscopy showed that, on 1 day of anaerobic culture, the tegument retained its integrity in the control worms (Fig 5A), whereas partial swelling and destruction were evident in the lower part of the tegument in the worms on day 1 after anaerobic culture (Fig 5B). Further ultrastructural examination of IACS-treated worms showed mitochondria with partial disappearance of the outer membrane and cristae on day 1, regardless of whether they were cultured aerobically or anaerobically. In the control group supplemented with DMSO, the mitochondrial structure was well maintained in both anaerobic and aerobic environments (Fig 6).

## Discussion

Mitochondria were isolated from *S. proliferum*, and the activities of its respiratory chain enzymes were first investigated to characterize the fundamental biochemical properties of this parasite. Based on these findings, representative quinone-binding site inhibitors were subsequently screened to evaluate their inhibitory effects on the identified mitochondrial complexes. Finally, the most promising inhibitor, IACS-010759, was selected for an initial culture-based experiment to assess its direct impact on living parasites as a proof-of-concept. Investigations into the metabolic pathways and potential therapeutic targets of *S. proliferum* have been limited. However, the present biochemical analyses have shed light on previously unresolved aspects.

In the assessment of mitochondrial respiratory chain activity in *S. proliferum*, the enzymatic activities of complexes I through IV were measured, and the activity of NADH-fumarate reductase, a component of the anaerobic respiratory pathway, was measured concurrently. This indicates that the parasite possesses fumarate respiration, enabling adaptation to low-oxygen environments. Ideally, quinol-fumarate reductase activity (QFR) should also have been measured, but its evaluation was not possible due to the unavailability of rhodoquinone. Given that this parasite exhibits both fumarate (anaerobic) respiration and aerobic respiration, it is considered to possess hybrid-type mitochondria capable of efficiently switching respiratory chains depending on the oxygen environment, similar to *E. multilocularis* [10]. Owing to the hybrid nature of the mitochondrial respiratory chain, effective eradication necessitates the concurrent inhibition of both the aerobic and anaerobic respiratory chains, specifically fumarate respiration. Consequently, this requires a compound capable of inhibiting either complex I alone or both complexes II and III simultaneously (Fig 2).

Quinone binding site inhibitors have been reported as anthelminthics [12–14]. The potent inhibition of complexes I, II, and III by rotenone, atpenin A5, and antimycin A, respectively, at low concentrations demonstrates that the present enzyme activity assay system is functioning correctly and indicates that each complex is indeed active and present in the enriched mitochondrial fraction. Although these compounds cannot be used in vivo due to their well-documented toxicity in mammals, their potent and specific inhibitory activities against each respective complex make them valuable starting points for the future development of safer derivatives.

Ascofuranone is a natural compound isolated from filamentous fungi and serves as a quinone-binding site inhibitor used in studies on *Trypanosoma* and *Echinococcus* [12,18,19]. Ascofuranone was found to concurrently inhibit both complexes II and III in *S. proliferum*. Though its IC₅₀ is not equivalent to that of atpenin A5, an ascofuranone (AF) derivative notably demonstrated inhibitory effects on complexes II and III at low concentrations. It has been reported that ascofuranone simultaneously inhibits mitochondrial complexes II and III in *E. multilocularis* [12], indicating that it also functions as a dual inhibitor targeting both the aerobic respiratory pathway and the fumarate respiratory pathway in *S. proliferum*. Atovaquone demonstrated potent inhibition of *S. proliferum* complex III at a low concentration, with an IC₅₀ value of 2.2 nM. In contrast, its inhibitory effect on complex II was minimal, indicating a limited capacity to suppress the anaerobic respiratory chain (fumarate respiration). As a result, atovaquone may exhibit only partial efficacy in eliminating parasites that rely on anaerobic respiration, thereby restricting its overall therapeutic potential. This limited impact on hybrid mitochondria has been corroborated by treatment studies conducted in *Echinococcus*-infected mouse models [10]. Nonetheless, whereas atovaquone exhibits a well-established long-term safety profile as an antimalarial agent, its clinical efficacy against *S. proliferum* infection remains unverified. Therefore, further *in vivo* and clinical studies are warranted to evaluate whether it can serve as a practical therapeutic option, especially in scenarios where alternative therapies are unavailable. The complex I inhibitor rotenone displayed strong inhibitory effects; however, its high toxicity in mammals limits its applicability in in vivo studies. Conversely, IACS-010759 has been successfully used in in vivo experiments as an anticancer compound [20,21]. This agent exhibits strong inhibitory potency against complex I, comparable to that of rotenone. Furthermore, since complex I serves as the common entry point for both aerobic and anaerobic respiratory chains, potent and selective inhibition of this complex was expected to induce distinct morphological changes in the parasite. For these reasons, IACS-010759 was selected for the culture-based experiment and its effects on *S. proliferum* larvae were assessed. However, it should be noted that IACS-010759 has encountered practical challenges in clinical development due to reported adverse effects, including peripheral neuropathy, which have limited its further clinical implementation [20]. This context underscores the importance of continuing to explore alternative complex I inhibitors with improved safety profiles for future therapeutic development.

The effect of IACS-010759 was more pronounced in aerobic culture. IACS-010759 is an inhibitor of complex I, which initiates both aerobic and anaerobic respiratory chains, and its impact is considered significant. In the present study, the control DMSO treatment showed no changes in either aerobic or anaerobic conditions throughout the experimental period, whereas IACS-010759 treatment caused significant damage to the worms in aerobic culture. This suggests that inhibition of complex I in aerobic culture is more damaging to worms or their mitochondria.

We hypothesize that the alternative cytoplasmic respiratory metabolism in tapeworms appears less able to compensate for complex I impairment in *S. proliferum* under aerobic conditions. In fact, previous studies have demonstrated that *Caenorhabditis elegans* can tolerate complex I impairment under hypoxic conditions, whereas such impairment becomes fatal under aerobic conditions. It has been suggested that this phenomenon is associated with the disruption of alternative cytoplasmic metabolic pathways and the generation of reactive oxygen species (ROS) in the presence of oxygen [22,23]. Loss of the outer membrane and cristae was observed in both cultures on day 1 after the start of culture. In an *Echinococcus* study, morphological changes in mitochondria have been observed in co-culture with several drugs [24–26], suggesting that the changes observed in the present study are likely a direct effect of the drug treatment. Indeed, since they were observed one day after the start of culture, IACS-010759 may have acted directly on the mitochondria of the worm, causing morphological changes in the outer membrane and cristae. Microscopically, swelling was observed in the lower layer of the tegument. This is thought to contribute to the roughness of the body surface during cultivation. However, although macroscopic observation of the parasites in the anaerobic medium on Day 1 showed no apparent signs of body surface disruption, histological examination under microscopy confirmed structural damage to the body surface. This discrepancy suggests that visual inspection alone may underestimate the actual damage level of the parasites. This is a critical caveat that must be considered when attempting to quantify parasite damage in future studies.

Although the relationship with mitochondria is unclear, structural deformation also occurred one day after the start of cultivation, suggesting a direct causal relationship. However, the swelling and collapse of the parasite observed by day 14 may indicate the disintegration course of the worm due to damage to organelles, rather than representing changes specific to IACS-010759.

It has been demonstrated that clarithromycin treatment targeting the mitochondrial ribosome in adult *E. multilocularis* results in friable body surfaces, vacuole formation, and swelling macroscopically and mitochondrial condensation and disappearance of cristae ultra-microscopically [26], supporting the present findings. It should be emphasized that the culture-based findings in the present study represent preliminary, proof-of-concept observations, designed to demonstrate for the first time whether observable morphological and histological changes could be induced in *S. proliferum* after compound administration, rather than to provide a fully quantitative or statistically powered analysis. The observed differences between aerobic and anaerobic conditions should therefore be interpreted as qualitative and exploratory in nature. Although structural disruption of the parasite bodies was observed in histopathological and morphological analyses, the development of reliable evaluation methods is necessary to verify complete elimination of the parasites as the next step.

### Limitations and future directions

A fundamental limitation of this study is the exclusive use of a single laboratory strain maintained by serial passage since 1981, which precludes direct comparison with wild-type isolates. Given the extremely rare ecological nature of *S. proliferum*, the collection of fresh wild isolates is practically impossible. Although long-term serial passage has been reported to alter pathogen virulence, infectivity, and pathogenicity [27], other studies have demonstrated that high levels of genetic diversity are maintained in long-term laboratory strains of *Schistosoma* and *Toxoplasma* [28,29]. Given these conflicting findings, one cannot exclude the possibility that the physiological characteristics of the currently maintained laboratory strain, including drug susceptibility and mitochondrial activity, may have diverged from those of naturally occurring wild isolates. With respect to the biochemical analyses, the enriched mitochondrial fraction yielded approximately 1 mg of protein per 5 g wet weight of worm tissue, representing the most significant physical constraint of this study. Due to this limitation, it was not feasible to use the multi-concentration experimental design required for nonlinear sigmoidal curve fitting, and IC₅₀ values were therefore determined by linear interpolation using concentrations bracketing the 50% inhibition threshold. Furthermore, due to the same physical constraints, a broader screening involving alternative pharmacological agents warrants further investigation in future studies. Regarding the in vitro culture-based drug evaluation, the experiments were designed not to quantify parasite survival rates, but rather to serve as a proof-of-concept assessment of morphological and histological changes induced by compound administration. *S. proliferum* is a large, branching organism reaching 1–6 cm in length, and unlike nematodes or cultured cells, accurate quantification by absorbance or fluorescence measurement using a microplate reader is not physically feasible. This parasite exhibits virtually no spontaneous motility in culture and possesses unique biological characteristics, including the absence of a mouth, anus, and digestive tract, proliferating instead by branching and budding. Consequently, localized and asynchronous necrosis occurs frequently within a single individual, where necrotic and viable regions coexist, making binary classification of viability inherently challenging. Based on the morphological changes identified in the present study, we intend to develop a quantitative system capable of assessing parasite viability and tissue damage in future investigations. In addition to the methodological constraints described above, several broader challenges remain to be addressed before the mitochondrial respiratory chain can be translated into a clinically viable drug target. Though the present in vitro biochemical experiments identified candidate compounds, their specificity against mammalian mitochondrial complexes and their toxicity in human cells must be carefully evaluated in future studies to ensure host safety. In addition, validation using animal models will be an indispensable next step, not only to confirm in vivo efficacy, but also to determine whether the tissue damage induced by prolonged treatment is entirely irreversible, or whether the parasite could regenerate and re-establish homeostasis from surviving tissue remnants.

## Conclusion

*S. proliferum* mitochondria are of a hybrid type, supporting both aerobic (complexes I–IV) and anaerobic (NADH-fumarate reductase) respiration to adapt to oxygen conditions. The quinone-binding site inhibitors IACS-010759, ascofuranone, and atovaquone inhibited mitochondrial complexes I, II, and III, respectively, with IC₅₀ values in the low nanomolar range. IACS-010759 induced mitochondrial damage and worm collapse in culture assays. These findings suggest that the mitochondrial respiratory chain of *S. proliferum* may represent a viable drug target for the development of novel therapeutic agents against this otherwise untreatable infection.

## Supporting information

S1 Data Underlying data for Tables 1 and 2. (XLSX)
